# Adjuvant A-PRF Application in Patients with Various Stages of Medication-Related Osteonecrosis of the Jaw (MRONJ): Case Series

**DOI:** 10.3390/reports8040240

**Published:** 2025-11-20

**Authors:** Irina Vasiļčenko, Ingrīda Čēma, Ieva Bāgante

**Affiliations:** 1 Department of Oral and Maxillofacial Surgery and Oral Medicine, Riga Stradiņš University, 16 Dzirciema Str., LV-1007 Riga, Latvia; 2 Centre of Oral and Maxillofacial Surgery, Pauls Stradiņš Clinical University Hospital, 13 Pilsoņu Str., LV-1002 Riga, Latvia

**Keywords:** advanced platelet-rich fibrin, A-PRF, bisphosphonate-associated osteonecrosis of the jaw, bone disease, growth factors, osteonecrosis of the jaws

## Abstract

**Background and Clinical Significance:** Medication-related osteonecrosis of the jaw (MRONJ) is a severe complication of antiresorptive therapies such as Zolendronic acid, used for bone metastases. Its management remains challenging, with outcomes often unpredictable. Platelet-rich fibrin (PRF), rich in growth factors, has been proposed as a potential adjunct to surgical treatment, aiming to promote tissue regeneration and improve patient outcomes. **Case Presentation:** We reported three clinical cases of MRONJ in patients previously treated with Zolendronic acid. All patients underwent surgical sequestrectomy combined with A-PRF application. Disease stages ranged from early to advanced (stages I–III). The success of treatment was evaluated based on how well the tissue healed, the extent of bone recovery, the amount of pain relief, and improvements in the patient’s quality of life. The patient with early-stage MRONJ achieved complete healing. On the other hand, the patients with stage II and III disease showed only partial clinical improvement. Nevertheless, all cases demonstrated significant subjective reduction in pain and enhanced overall quality of life following PRF therapy. **Conclusions:** Early-stage intervention offers the best prognosis for MRONJ. While A-PRF may improve postoperative comfort and quality of life, its curative effect appears limited in advanced disease. This highlights the need for further randomized clinical trials to demonstrate the role of A-PRF in the treatment of MRONJ.

## 1. Introduction and Clinical Significance

Medication-related osteonecrosis of the jaw (MRONJ) is a condition in which areas of exposed bone appear in the jaw and fail to heal within eight weeks in patients who have a history of antiresorptive therapy [1]. This study is particularly relevant because bisphosphonates are widely used in the treatment of patients with proven bone metastases, multiple myeloma, and Paget’s disease. Osteonecrosis of the jaws is an atypical form of osteomyelitis. Cases of atypical osteonecrosis were first described in 2003 by Marx [2]. Currently, the reported incidence of osteonecrosis of the jaws varies across different studies; it can range from 1% to 15% in patients receiving parenteral bisphosphonates for bone metastases [3,4]. Earlier reports and small case series occasionally cited wide ranges (up to 15%) for MRONJ incidence in high-risk oncologic cohorts receiving long-term antiresorptive therapy [5]. However, large-scale prospective evidence from the SWOG S0702 trial, which followed 3491 patients with bone metastases treated with intravenous ZA, demonstrated a cumulative MRONJ incidence of 0.8% after one year, 2.0% after two years, and 2.8% after three years (same reference). Comparable frequencies (~2.8%) were confirmed in the analysis by Brunner C. et al. [6], while a 2025 meta-analysis by Kujanpää M. [7] reported an overall range between 0.4% and 2.3%, depending on dosage, treatment duration, and local risk factors. The previously cited 1–15% range refers to mixed-population estimates, while the real-world incidence of MRONJ among oncologic patients receiving intravenous ZA generally remains below 3%, rising gradually with prolonged therapy and poor oral health status.

**Figure 1:**
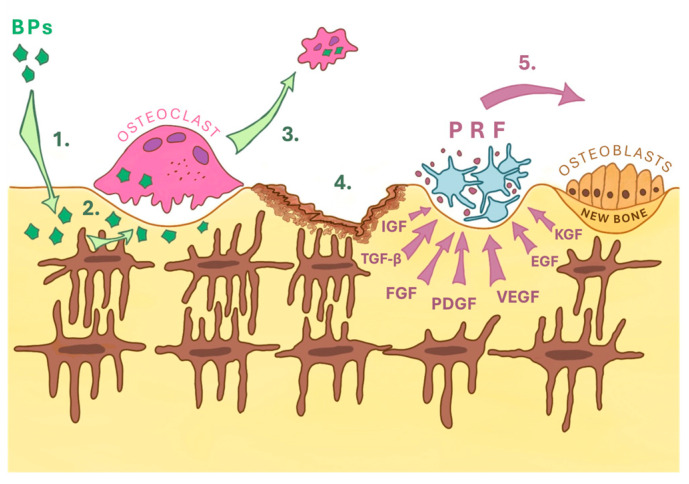
The mechanisms of bisphosphonates and PRF action (by I.Vasiļčenko). 1. Bisphosphonates penetrate the bone. Due to their affinity with hydroxyapatite, bisphosphonates are incorporated into its crystals. Bisphosphonates (BPs) are accumulated and retained in the bone matrix. 2. Hydroxyapatite crystals are broken down by osteoclasts through pinocytosis. During resorption, BPs are incorporated into osteoclasts through transcytosis. Bisphosphonates that enter the osteoclast reduce its activity. 3. BPs induce apoptosis of osteoclasts. 4. Osteonecrosis zone of bone. 5. PRF contains growth factors and stimulates the development of new bone. IGF, Insulin Growth Factor; TGF-β, Transforming Growth Factor Beta; FGF, Fibroblast Growth Factor; PDGF, Platelet Derived Growth Factor; VEGF, Vascular Endothelial Growth Factor; EGF, Epithelial Growth Factor; KGF, Keratinocyte Growth Factor.

Bone metastases seriously worsen the course of the disease as they cause the occurrence of pain syndrome, the threat of pathological fracture, dysfunction of the limbs, etc. This is why the fight against bone metastases is so important. Treatment of bone metastases involves inhibiting the activity of osteoclasts and their precursors. Bisphosphonates are the most active inhibitors of osteoclasts and subsequent bone resorption. The mechanism of all bisphosphonates action is similar—bisphosphonates penetrate bone tissue, interact with hydroxyapatite crystals, concentrate around osteoclasts, and disrupt the formation of the cytoskeleton in osteoclasts. As a result, the migration of osteoclasts, their resorptive capacity, and the adhesion of tumor cells are inhibited (see Figure 1).

It is important to understand that due to the increase in bone strength, the bone’s ability to regenerate decreases. Osteonecrosis of the jaws occurs due to the peculiarities of its structure. Both the upper and lower jaws are very vulnerable to bacterial infection since the mucous membrane covering the jaws is very thin and sensitive. The infection spreads directly from infected teeth and infected periodontal pockets. Also, the infection enters through microtraumas of the oral mucosa, which occurs because of the pressure of the dentures on the denture bed and bone exostoses [8,9,10]. Local cytotoxicity of bisphosphonates increases in an acidic environment under conditions of abnormal jaw remodeling and reduced angiogenesis [11].

Currently, the most widely used classification of MRONJ is that proposed by the American Association of Oral and Maxillofacial Surgeons (AAOMS) in 2009. In stage 0, patients have no clinical evidence of necrotic bone but may present with non-specific symptoms or radiological findings. These may include odontalgia not attributable to an odontogenic cause, the loosening of teeth without chronic periodontal disease, intraoral or extraoral swelling, radiographic evidence of alveolar bone loss or resorption not related to periodontal disease, and areas of osteosclerosis involving the alveolar and/or basilar bone. In stage I, patients present with exposed and necrotic bone or a fistula that probes into the bone but remain asymptomatic and show no signs of infection or inflammation. Radiographic findings described in stage 0 may also be observed, though they are localized to the alveolar bone region. In stage II, patients present with exposed and necrotic bone or a fistula that probes into the bone, accompanied by clinical signs of infection or inflammation. These patients are symptomatic. Radiographic findings may include a mixed pattern of diffuse osteosclerosis and osteolysis of the jawbone extending from the alveolar region, the thickening of the mandibular canal, a periosteal reaction, maxillary sinusitis, and sequestrum formation. In stage III, patients present with exposed and necrotic bone or a fistula that probes into the bone, accompanied by infection and at least one of the following: extension of necrosis to the inferior border and ramus of the mandible, the maxillary sinus, or the zygoma; pathologic fracture; extraoral fistula; oral–antral or oral–nasal communication; or osteolysis extending to the inferior border of the mandible or the floor of the sinus [10,12].

Several methods of treating MRONJ have been described in the literature, but none of them have provided convincing stable treatment results. However, the application of A-PRF as an adjunct to surgical sequestrectomy has shown promising results in some studies. In a study using PRF, de Almeida Barros Mourão et al. (2020) [13] reported complete treatment success. For L-PRF, Zelinka et al. (2021) [14] achieved success rates of 85–94%, Özalp et al. (2021) [15] reported 69%, Aslam et al. (2024) [16] observed 100%, and Yalcin-Ülker (2023) [17] reported an 80% success rate. Therefore, our clinic has also started to use the A-PRF technique in addition to the treatment of various stages of MRONJ.

The aim of this article is to present three clinical cases of MRONJ, managed surgically through sequestrectomy, with the adjunctive use of advanced platelet-rich fibrin (A-PRF). Considering its angiogenic, antibacterial, and regenerative properties, A-PRF may offer potential benefits as a supportive treatment for MRONJ. Our clinical observations suggest that A-PRF application could positively influence the course of jaw osteonecrosis by promoting tissue repair and regeneration; however, this hypothesis requires validation in larger, controlled studies.

## 2. Case Presentation

### 2.1. The First Case (See Table 1)

**Figure 2:**
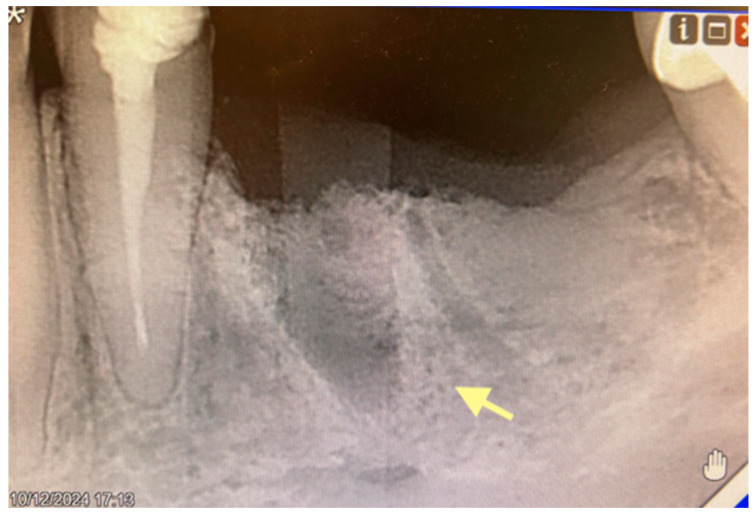
Initial signs of osteonecrosis of the lower jaw in the area of the tooth 44 alveola (12 October 2024).

The first patient was a 64-year-old female with bone metastases (parietal bone) in 2019, but osteonecrosis of the mandible developed after tooth 34 extraction in May 2024 (see Figure 2).

**Table 1:** Summary of data from three clinical cases.

	The First Clinical Case	The Second Clinical Case	The Third Clinical Case
Demographics	64-years old female	82-years old female	75-years old female
Primary disease	Malignant breast tumor (IV st.); bones metastases	Malignant breast tumor (IV st.); bones metastases	Malignant breast tumor (IV st.); bones and lung metastases
Comorbidities	-	-	-
Bad habits	Non-smoker Non-alcoholic person	Non-smoker Non-alcoholic person	Non-smoker Non-alcoholic person
Risk factors (periodontal disease)	-	dd13,14,16,17, 22,23,38,41.42.31.32 Chronic periodontitis with deep bone pockets	Adentia of the upper jaw; hypodentia of the lower jaw; dd 32,33; Chronic periodontitis
Oral hygiene	Good	Satisfactory	Satisfactory
Drug exposure history	Zolendronic acid 5 mg (i/v) once a month, 2021.g. (March)–024.g. (November)	Zolendronic acid 5 mg (i/v) once a month, 2021.g. (January)–2024.g. (June)	Zolendronic acid 5 mg(i/v) once a month—2021.g. (August)
Clinical finding	MRONJ in reg.dd34-35 STAGE I	MRONJ in reg.dd45-46 and reg.dd41-31 STAGE II	MRONJ in reg.dd21-28, dd34-36, dd31-46 STAGE III
Surgery	24 February 2025 Sequestrectomy of the lower jaw (reg.dd34-35) and A-PRF application	5 May 2024 Sequestrectomy of the lower jaw (reg.dd45-46) and A-PRF application. 4 April 2025 Sequestrectomy of the lower jaw (reg.dd45-46, reg.dd41-31) and A-PRF application	25 September 2023 Sequestrectomy of the lower jaw (III quadrant) and of the upper jaw (II quadrant) 19 February 2024 e/o incision; revision of submandibular abscess 17 December 2024 Sequestrectomy of the lower jaw (IV quadrant) 20 March 2025 Sequestrectomy of the lower jaw (IV quadrant) and of the upper jaw (II quadrant); A-PRF application
Outcome	Complete healing	Surgical wound on the lower jaw in reg.dd 45-47 healed completely, but closed partially in reg.dd 41-31	Surgical wounds reg.dd32-42 and reg.dd27-28 have not healed, but surgical wounds reg.dd34-46 healed completely
Microbiology	Normal microflora of the upper respiratory tract grows in the culture	Streptococcus constellatus (sensitive to Penicillin, Cefotaxime, Ceftriaxone, Clindamycin, Vankomycin)—5 May 2024. Porphyromonas gingivalis, Parvimonas micra, Prevotella buccae—7 April 2025.	Streptococcus constellatus (sensitive to Penicillin, Cefotaxime, Ceftriaxone, Clindamycin, Vankomycin)—27 January 2025 Streptococcus constellatus Actinomyces odontolyticus Bacteroides thetalomicron Prevotella nigrescens—21 March 2025.
Morphological picture	The morphological picture corresponds to small bone fragments; soft tissue with focal intense lymphomacrophage inflammatory infiltration	The morphological picture corresponds to granulation tissue of varying degrees of maturity, with chronic purulent inflammation	The morphological picture corresponds to bone fragments with necrosis; neutrophilic leukocyte infiltration
Antibacterial therapy	4 days in hospital—Amoxiclav 1.2 g 3x(i/v); after that, continued T.Amoxyclav 1 g 2x(p/o) for 4 days	4 days in hospital—Amoxiclav 1.2 g 3x(i/v); after that, continued T.Amoxyclav 1 g 2x(p/o) for 7 days (each case)	8 days in hospital—Amoxiclav 1.2 g 3x(i/v); after that, continued T. Amoxyclav 1 g 2x(p/o) for 10 days (February 2024) 5 days in hospital—Amoxiclav 1.2 g 3x(i/v); after that, continued T.Amoxyclav 1 g 2x(p/o) for 7 days (December 2024) 6 days in hospital—Dalacin 0.6 g-2x (i/v); after that, continued caps. Dalacini 0.3 g-3x (p/o)
Follow-up	The first visit is a week after the operation; then, once a month; the last visit is 30 June 2025, when complete healing is confirmed	The first visit is a week after the operation; then, once a month; last visit is 29 September 2025 (complete healing, patient uses new partial plate for lower jaw)	The first visit is a week after the operation, then as needed, but no less than once a month; the last visit was 6 October 2025

According to the classification of osteonecrosis of the jaws, the patient had stage I. Despite antibacterial therapy, an intraoral fistula formed. Therefore, in February 2025, an operation was performed (sequestrectomy with subsequent A-PRF placement).

**Figure 3:**
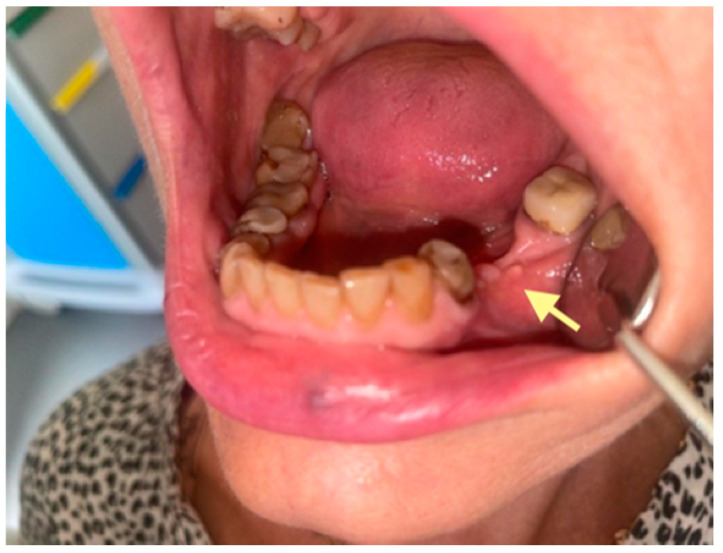
Four months after operation (30 June 2025). Complete heeling is noted.

After the operation, complete healing was noted (see Figure 3). Although the patient continues to receive chemotherapy for the underlying disease, osteonecrosis is currently not observed. Most likely, the reason for the successful treatment is that the process was treated at an early stage.

### 2.2. The Second Case (See Table 1)

**Figure 4:**
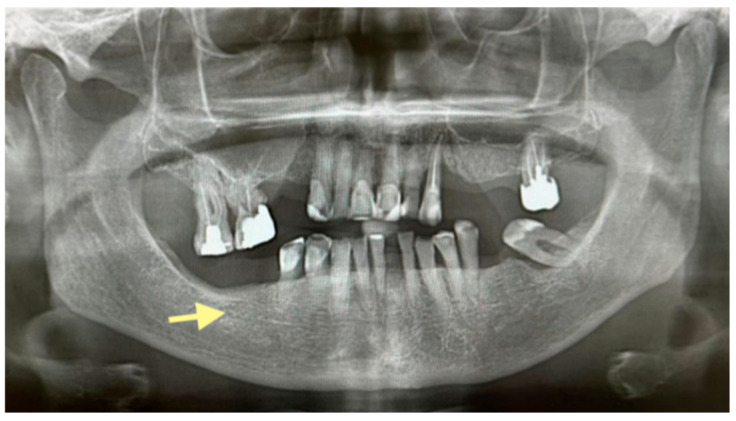
OPG (orthopantomogram) before surgery (12 January 2024). In the fourth quadrant, we do not see any changes, which are typical in the initial stages of osteonecrosis.

**Figure 5:**
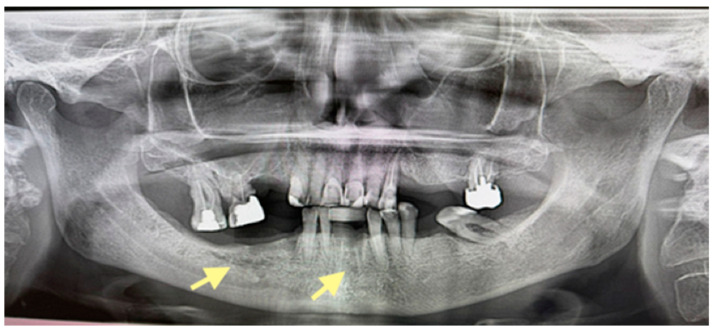
OPG (orthopantomogram) performed on 21.08.2024.

**Figure 6:**
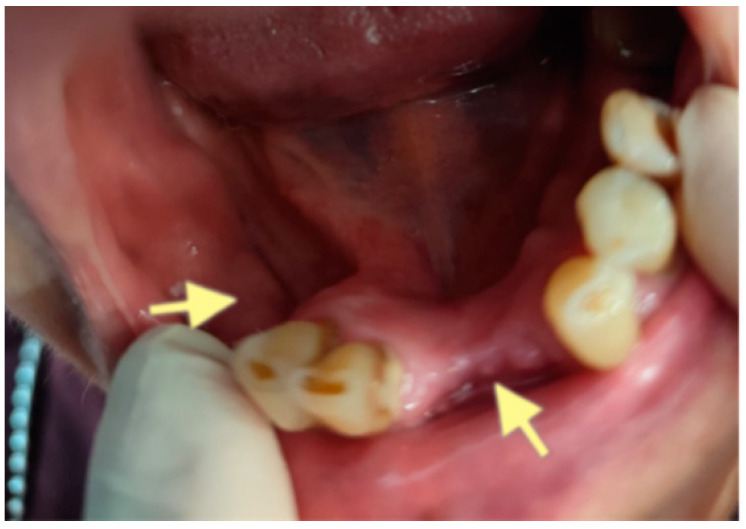
Control visit of the patient after the second surgery, one month after the second operation performed on 28 May 2025.

The second patient was an 82-year-old female with osteonecrosis of the mandible in the fourth quadrant, which developed after the removal of tooth 46 on November 2023 (see Figure 4). The patient had second-stage osteonecrosis of the jaw. Initially, the patient underwent a revision of the socket of the extracted tooth and was prescribed antibacterial therapy. Despite this treatment, the patient continued to complain of a non-healing wound on the lower jaw in the area of the molars with periodic purulent discharge. In addition, these complaints were supplemented by a complaint of an intraoral fistula on the lower jaw in the area of the frontal teeth. Therefore, in May 2024, the patient underwent surgery (sequestrectomy and application of A-PRF). After the operation, the patient’s condition improved, purulent discharge from the wound stopped, the pain ceased, and the postoperative wound closed completely (see Figure 4). However, after a month, dehiscence of the wound was noted. Therefore, it was decided to repeat the operation, performed on 4 April 2025, and, in addition, to expand the scope of the surgical procedure, the sequestrectomy of the lower jaw was performed not only in the region of dd45-46 but also in the central area, simultaneously applying A-PRF. As a result of the treatment, the surgical wound on the lower jaw in the area of the molars on the right healed completely, but in the area of the incisors, it closed partially (see Figure 5 and Figure 6).

However, on 29 September 2025 (last control visit), we noticed complete healing in both areas of the lower jaw. The patient could use a new partial plate for the lower jaw.

### 2.3. The Third Case (See Table 1)

**Figure 7:**
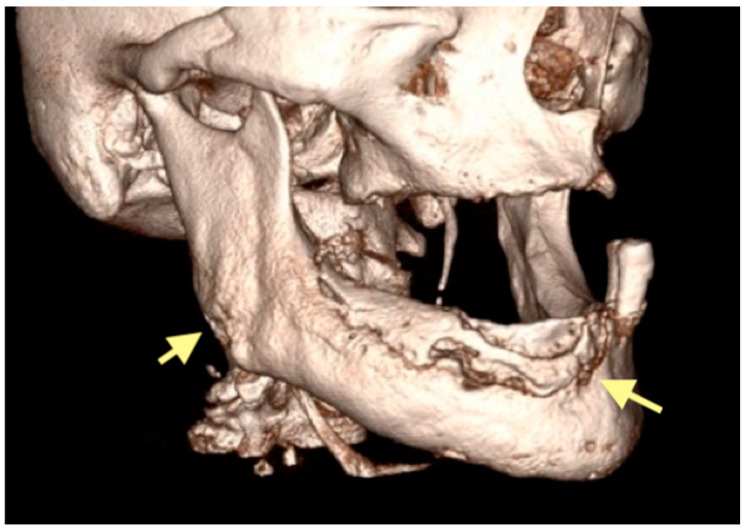
CT of the head and facial bones (16 December 2024).

**Figure 8:**
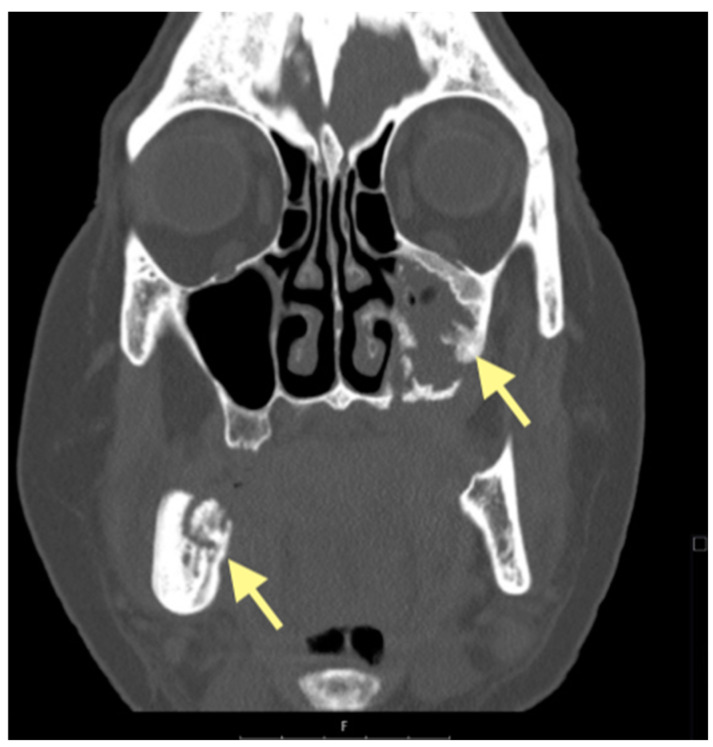
CT of the head and facial bones (16 December 2024).

**Figure 9:**
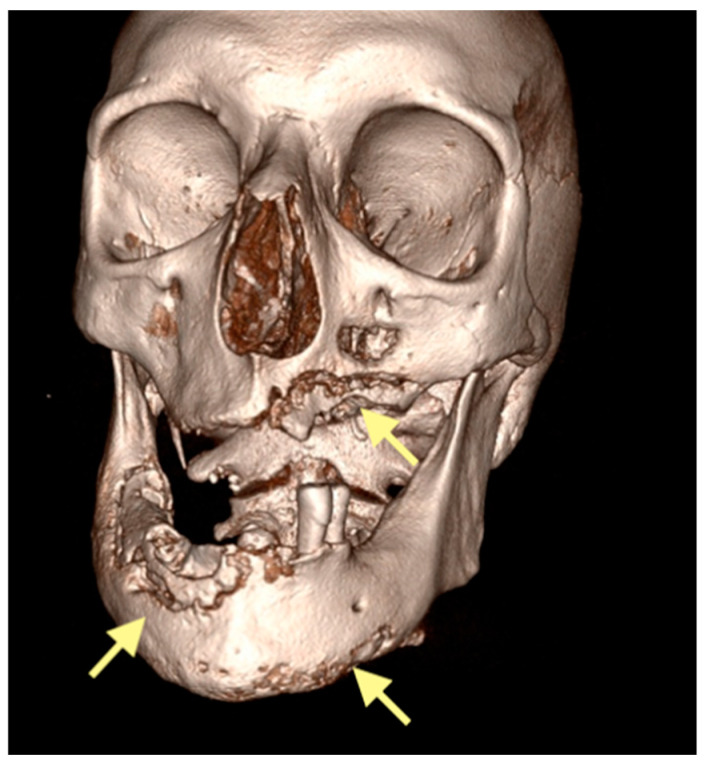
CT of the head and facial bones (16 December 2024).

**Figure 10:**
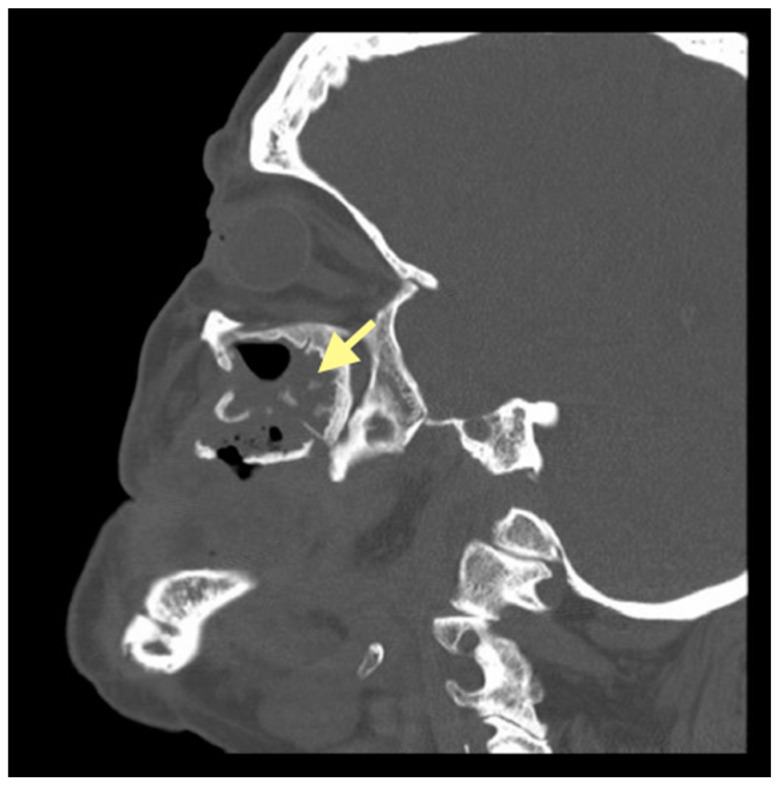
CT of the head and facial bones (19 March 2025) before the last operation (sequestrectomy of the lower and upper jaws; antrotomy performed).

**Figure 11:**
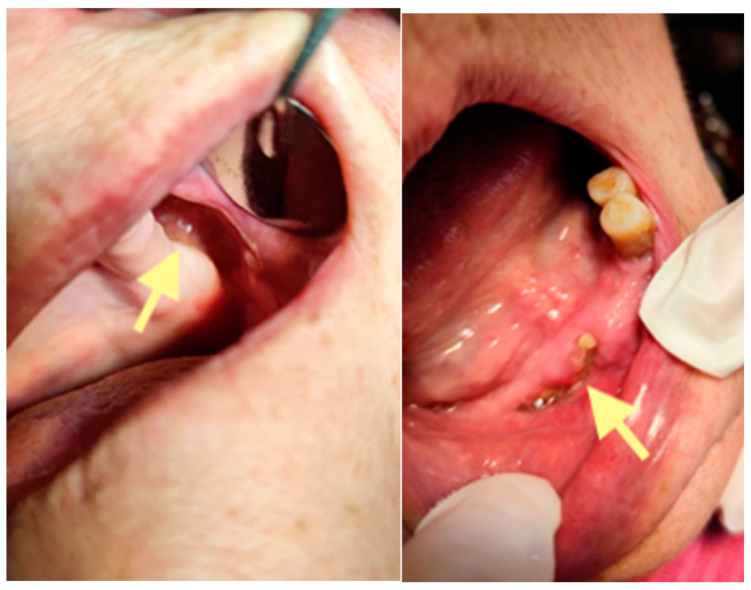
The photos show the patient 2 months after the last operation (sequestrectomy of the lower and upper jaws; antrotomy and A-PRF application). The healing of the surgical wounds is incomplete, but there is a positive tendency towards healing.

The third patient was a 75-year-old female with osteonecrosis of the mandible and maxilla in the fourth and in the second quadrant (see Figure 7, Figure 8 and Figure 9). The patient had third-stage jaw osteonecrosis. In 2023, the patient was diagnosed with osteonecrosis of the upper and lower jaw caused by antiresorptive therapy (until August 2021, the patient regularly used *Zolendronic acid*; then, this drug was replaced by the RANKL inhibitor *Denosumab*). The treatment was periodically complicated by submandibular and submental abscesses. In addition, due to the progression of the underlying disease, constant courses in chemotherapy were administered. The patient underwent three operations, which included sequestrectomy of the jaw with simultaneous use of A-PRF, antrotomy with revision of the maxillary sinus, closure of the oroantral communication, and opening and revision of abscesses of the soft tissues (see Figure 10 and Figure 11)

Despite such an unfavorable disease course, it was possible to achieve an improvement in the patient’s condition. Her pain in the upper and lower jaw has significantly decreased, and the regular bleeding from inflamed gums has stopped.

### 2.4. The Duration of Postoperative Follow-Up Varied Among Cases

Case 1: Observed for 4 months after the final intervention, showing stable mucosal coverage and absence of exposed bone or pain.

Case 2: Initially followed for 6 weeks, and on the additional follow-up visit on the 29 September 2025, complete and stable mucosal healing was confirmed.

Case 3: Initially reviewed for 8 weeks, and during the 6 October 2025 control, partial non-healing persisted in the region of tooth 28, and mucosal fragility remained in the 31–45 region.

No new signs of infection or bone exposure were noted in any case.

## 3. Discussion

The study of the problem of the occurrence of such serious complications as medication-related osteonecrosis of the jaw (MRONJ), along with the development of a management strategy and the prevention of this pathology, is a great challenge in maxillofacial surgery.

Jaws osteonecrosis is a side-effect of antiresorptive therapy, which, in our study, was due to bone metastasis. The exact mechanism underlying MRONJ remains unclear. Several factors appear to contribute to its development, including impaired bone remodeling, local inflammation or infection, reduced angiogenesis, and immune system dysfunction [18]. As a result, there is a chronic process, with exposed bone accompanied by the impairment of remodeling and healing. A-PRF is an autologous, non-allergenic, and non-immunogenic platelet-rich preparation derived from blood and enriched with growth factors. These growth factors promote healing and help reduce postoperative complications. Wound repair is further supported by their gradual release, lasting up to four weeks, which stimulates regenerative processes such as cell proliferation and differentiation—particularly of fibroblasts, endothelial cells, and osteoblasts—that are essential for both soft- and hard-tissue regeneration. In addition, A-PRF enhances neovascularization, modulates inflammation, and accelerates the formation of epithelial and connective tissues [19,20].

Only a limited number of studies have examined the use of PRF as an adjunctive treatment for MRONJ. Most of the available evidence comes from case series and retrospective studies [21,22,23]. Two prospective studies without control groups reported disease resolution rates of 93% and 77% in patients treated with surgery combined with PRF [24,25]. In studies evaluating A-PRF+ (as distinct from A-PRF), Giudice et al. (2020) [26] reported that 74.5% of their study population were oncology patients, with stage II cases (53%) being slightly more common than stage III cases (47%) according to the AAOMS classification. Their results showed that A-PRF+ improved patients’ quality of life during the first month after surgery by reducing pain and lowering the risk of postoperative infections. However, this effect was not maintained in longer follow-up periods. Similarly, in a study by Blatt et al. (2022) [27], investigating the impact of A-PRF on the healing of osteonecrotic defects in cancer patients, no statistically significant association was observed between the use of A-PRF and the healing process. Therefore, there is no clear evidence of a direct relationship between wound healing and the use of A-PRF [27,28]. In our review of three clinical cases, complete recovery occurred in patients with early-stage osteonecrosis, while partial recovery occurred in patients with stage II and III. It is logical to assume that the results of treatment directly depend on how early the treatment is started. A significantly better response to A-PRF treatment was observed in patients with smaller lesions. In the first stage of osteonecrosis of the jaws, the damage to the bone is small and there are no signs of infection, unlike later stages of osteonecrosis (i.e., the second and third), when infection sets in and osteonecrosis spreads further and occupies significant parts of the jaw. Therefore, healing in the first stage occurs faster, with a lower risk of relapse. In contrast, one patient experienced progression of the underlying neoplastic disease, which led to a decline in overall health and may have interfered with the healing process.

We must consider that oral microflora also play an important role in MRONJ development. In the third case, the most complicated case in our microbiological findings was *Actinomyces odontolyticus* (an anaerobic, Gram-positive bacterium). Necrotic bone in MRONJ lesions is frequently colonized by *Actino myces* species, which are commensal microorganisms of the oral cavity. The onset of infection and actinomycosis is often linked to impaired host immunity, a condition commonly observed in patients with oncological diseases. Areas of bone necrosis provide an anaerobic microenvironment that favors the proliferation of these bacteria. In addition to *Actinomyces*, other microorganisms isolated from MRONJ lesions include *Streptococcus constellatus* (Gram-positive, anaerobe cocci), *Parvimonas micra* (Gram-positive anaerobe cocci), *Porphyromonas gingivalis* (Gram-negative, rod-shaped, anaerobic bacteria), and *Prevotella buccae* (Gram-negative, rod-shaped, anaerobic bacteria).

Microbiological examination across follow-ups revealed predominantly anaerobic polymicrobial flora typical of oral biofilm communities. In the first case, cultures showed normal upper respiratory tract microflora and *Streptococcus constellatus* (sensitive to penicillin, cefotaxime, ceftriaxone, clindamycin, and vancomycin). In the second case, we identified a mixed anaerobic culture *Porphyromonas gingivalis*, *Parvimonas micra*, *Prevotella buccae*, and *S. constellatus*. In the third case, a mixed anaerobic flora was again identified: *S. constellatus*, *Actinomyces odontolyticus*, *Bacteroides thetaiotaomicron*, and *Prevotella nigrescens*. Histopathology showed necrotic lamellar bone colonized by filamentous bacteria consistent with *Actinomyces* spp., along with granulation tissue and chronic inflammatory infiltrate. The repeated isolation of *S. constellatus* (member of the *Streptococcus anginosus* group) and black-pigmented anaerobes (*Porphyromonas*, *Prevotella*) suggests a biofilm-mediated process sustained by microaerophilic and anaerobic niches within exposed bone. The persistence of *S. constellatus* and emergence of strict anaerobes (*Porphyromonas*, *Prevotella*, *Parvimonas*, *Bacteroides*) illustrate how biofilm-forming consortia can maintain low-grade infection and delay epithelial closure in MRONJ lesions. *S. constellatus*, although penicillin-sensitive, often coexists with β-lactamase–producing *Prevotella*, potentially diminishing antibiotic efficacy without mechanical debridement. The detection of *Actinomyces odontolyticus* further supports a chronic colonization pattern as this species adheres tightly to necrotic bone and resists superficial irrigation.

Accordingly, our management combined thorough debridement, local A-PRF placement to reduce anaerobic niches, and targeted short-course antibiotic therapy (penicillin or amoxicillin–clavulanate ± metronidazole). This integrative approach correlated with progressive epithelialization and the absence of exposed bone at ≥8 weeks.

Of course, we must not forget about regular dynamic monitoring of the patient since the progression of the underlying disease can worsen the condition of the jaws. MRON of the jaw, while well-documented in the literature, is a rare complication. As a result, it remains difficult to draw definitive conclusions from the available literature and to assess the preventive potential of PRF. PRF should not be considered a cure-all; however, it often shows a favorable influence on healing outcomes Moreover, even in the most severe cases of widespread osteonecrosis, sequestrectomy and the use of PRF significantly improve the patient’s condition.

In this small descriptive series, the application of A-PRF was associated with improved local conditions, including pain reduction, decreased mucosal inflammation, and accelerated soft-tissue coverage in early-stage lesions. These short-term findings suggest a supportive and regenerative adjunctive role of A-PRF rather than a definitive curative effect. In advanced MRONJ, residual or partially unhealed areas persisted despite local improvement, underlining the complexity of these lesions and the multifactorial nature of healing. The biological plausibility of A-PRF—its capacity to provide growth factors, leukocytes, and matrix for angiogenesis—warrants further study under controlled conditions.

## 4. Materials and Methods

This article describes three clinical cases of the occurrence and development of jaw osteonecrosis (upper and lower jaw) in patients receiving antiresorptive therapy due to bone metastasis from breast cancer. In all patients, jaw osteonecrosis was caused by the use of *Zolendronic acid*, and it was primarily a chronic process that was diagnosed after long-term and unsuccessful treatment for alveolitis after tooth extraction. All patients received surgical treatment (sequestrectomy) and A-PRF application. During surgery, four 10 mL samples of venous blood were collected in sterile, anticoagulant-free tubes. The samples were then immediately centrifuged using a PRF Centrifuge Duo Quattro (PRF DUO by Choukroun (PRF Process, Nice, France)with rotor radius 11 cm, max RCF 2490 g) at 1300 rpm for 14 min. Then, the A-PRF clots were put under the press, and A-PRF membranes were made. The surgical procedures were performed under general anesthesia, with additional local infiltration using 4% *Articaini* solution *with epinephrine* (1:100,000) in the first and third cases, and local Sol. Articaini 4% *with epinephrine* (1:100,000) infiltration anesthesia in the second case. A No. 15c scalpel blade was used to raise a mucoperiosteal flap. The epithelialized mucosal margins were prepared and debrided. The necrotic bone was curetted, and the base of the lesion was debrided with a round bur under irrigation with 0.9% NaCl until the vital, bleeding bone was exposed. After that, four A-PRF membranes were placed on the bone. The wound was sutured with local mucoperiostal flaps without tension with Vicryl (3/0). The surgical material was sent for pathohistological and microbiological examination. During the pathohistological examination, in all cases, a clinical picture was established corresponding to osteonecrosis of the jaws (fragments of bone tissue with necrosis and infiltration of neutrophilic leukocytes). Microbiological examination showed the prevailing microflora (*Str. constellatus*, *Str. anginosus*, *Parvimonas micra*, *Bacteroides thetaiotaomicron*). Complex treatment, as well as the use of A-PRF, was analyzed. Patients attended weekly follow-up visits during the first month, followed by monthly appointments until the lesion had fully healed. For partially healing lesions (as in the third case), the frequency of follow-up visits was adjusted individually.

## 5. Conclusions

Our results varied: the patient with stage I underwent a complete recovery, but the others with more severe stages did not undergo a complete recovery. Although A-PRF improved postoperative comfort and quality of life, its curative effect appears limited in advanced disease. A-PRF improved patient-reported outcomes (pain, comfort, quality of life) but did not consistently induce complete bone healing in advanced MRON. Early-stage MRONJ may respond favorably to sequestrectomy combined with A-PRF; in advanced stages, A-PRF appears to improve patient-reported comfort but shows limited curative effect. In the future, controlled trials with validated outcomes and longer follow-up are required to define its therapeutic role. These findings underscore the need for further randomized clinical trials to clarify the role of A-PRF in the management of MRONJ.

## Data Availability

The data presented in this study are available on request from the corresponding author. The data are not publicly available due to patient data protection law and confidentiality.
